# Factors Associated With Time to Elimination of Porcine Epidemic Diarrhea Virus in Individual Ontario Swine Herds Based on Surveillance Data

**DOI:** 10.3389/fvets.2019.00139

**Published:** 2019-05-08

**Authors:** Amanda M. Perri, Zvonimir Poljak, Cate Dewey, John C. S. Harding, Terri L. O'Sullivan

**Affiliations:** ^1^Department of Population Medicine, Ontario Veterinary College, University of Guelph, Guelph, ON, Canada; ^2^Department of Large Animal Clinical Sciences, Western College of Veterinary Medicine University of Saskatchewan, Saskatoon, SK, Canada

**Keywords:** swine, porcine epidemic diarrhea virus, time to elimination, Ontario, surveillance

## Abstract

Porcine epidemic diarrhea virus (PEDV) emerged into Canada in January of 2014. The virus was considered to be of high importance and the number of new cases were tracked using different mechanisms by stakeholders such as veterinary services from the provincial government and the swine industry. In addition to the initial date of infection, veterinary organizations in the swine industry maintained a disease control program (DCP) database that contained the date of declaration of freedom from PEDV in individual herds. Such data allowed for the determination of the duration of PEDV infection in individual herds based on herd type, year and season of diagnosis. Therefore, the objective of this study was to determine time to PEDV elimination in Ontario swine herds infected between 2014 and 2017, on the basis of records from the DCP database; and to identify factors associated with the likelihood of elimination. Duration of time to eliminate PEDV was estimated using Kaplan-Meier survival curves. The final Cox's proportional hazard model included herd type, season and year of diagnosis. The hazard of PEDV elimination for premises that were farrow-to-wean was 3.36 times larger (*P*-value: 0.044, 95% CI: 1.03, 10.93) than for farrow-to-feeder herds. Herds diagnosed in the summer and fall had hazard ratios of 1.40 (*P*-value: 0.044, 95% CI: 1.03, 10.93) and 7.32 (*P*-value: <0.001, 95% CI: 3.12, 17.18), respectively compared to herds diagnosed in the winter months. The hazard ratio for herds diagnosed in 2015 was 0.54 (*P*-value: 0.015, 95% CI: 0.33, 0.89) compared to herds diagnosed in 2014. Factors associated with time to elimination are likely reflective of the complexity of infection control practices applied in herds with different demographics and population structures, seasonal variability in the pathogen transmissibility, and the availability of resources to manage an emerging production-limiting disease. The median times to elimination were relatively long, which could be due to how it was measured, decisions made at the level of individual herds or delays related to reporting PEDV elimination. Design of control measures for production-limiting diseases at the regional level should take these factors into consideration.

## Introduction

Porcine epidemic diarrhea virus (PEDV) emerged into Canada in January 2014, soon after the initial detection in the United States (1). The virus is highly contagious and is associated with mortality ranging between 80 and 100% in suckling pigs (2–4). Incursion of porcine epidemic diarrhea (PED) has a large impact on animal health and profitability of individual farms; which can result in high loses for the entire swine-producing sector when a large outbreak occurs (4, 5). Despite this, PED is not considered a reportable disease at the federal level in Canada, similarly to other jurisdictions. Nonetheless, it is considered as provincially reportable in several Canadian provinces including Ontario (6). Both, legislative framework in the province of Ontario and concerns about the impact of this disease in the swine-producing sector supported establishment of several mechanisms of PEDV surveillance with different surveillance coverage (7). One of the surveillance mechanisms is based on the disease control program (DCP) database, which is known as the PED Ontario Area Regional Control and Elimination program (ARC&E). The DCP is based on swine producer volunteer participation and was implemented to monitor disease trends over time. The uniqueness of the DCP database is that it tracks the dates of the initial PEDV incursion, as well as the dates the herds declare freedom from infection from PEDV on the basis of established criteria. This allowed detailed estimation of incidence and prevalence over time in this source population (7). Briefly, the estimated prevalence and 95% confidence intervals (CI) of the virus at the end of 2014, 2015, and 2016 were 4.36 (3.07, 5.99), 2.25 (1.49, 3.26), and 1.35 (0.79, 2.16), respectively (7). A decrease in prevalence, despite occurrence of new cases, has been achieved through implementation of targeted elimination programs at the individual herd level. Soon after PEDV emerged, veterinary practitioners developed approaches that allowed planned elimination of PEDV from swine herds. However, the time to elimination of the virus was premises-dependent and depended on the elimination strategy employed. For planning purposes, the time to PEDV elimination for specific herds could be projected on the basis of the herd type, its demographics, and infection control practices that are planned to be implemented. However, under field conditions, additional factors such as the demographics of the entire production system, the number of animal movements, availability of resources and the herd owners' overall willingness to eliminate a production-limiting disease could affect time to PEDV elimination for specific herds. Since the dates of disease incursion and elimination in individual herds are available, the DCP database could be an appropriate resource for evaluating the time to PEDV elimination under field conditions in the entire population (source population) participating in the DCP program. Therefore, the objective of this study was to determine time to PEDV elimination in Ontario swine herds infected between 2014 and 2017, on the basis of records from the DCP database; and to identify factors associated with the likelihood of elimination.

## Methods

### Data Source

The source population for this study was the OSHAB PED Ontario Area Regional Control and Elimination program (ARC&E) database. This DCP and database was initially created for controlling porcine reproductive and respiratory syndrome virus (PRRSV) (8) and then was adapted to include PEDV when it emerged into Canada in 2014. The DCP is a voluntary program that collects diagnostic data including PEDV herd status of Ontario swine herds as outbreaks are reported, or as herds are classified as having eliminated PEDV from premises. The data collected from the participating herds include the premises identification number, herd type, herd size, date of enrollment into the database, PEDV status of premises on date of enrollment and the date(s) in which the premises changed their PEDV status to “free-from-PEDV.” For a premises to be included in the current study the following inclusion criteria were fulfilled: (1) the premises participated in the DCP from January 2014 to October 2017, (2) the premises was located in Ontario, and (3) the PEDV infection status of the premises was available.

### Premises PEDV Infection Status

The DCP monitors infection status of the volunteer premises over time. Thus, the database contains herd (premises) infection status information i.e., whether a herd has eliminated the virus, whether any subsequent infection has occurred or any other changes in infection status, and the dates when the changes in infection status occurred. In the database, there are 4 types of premises infection status classifications: (1) confirmed positive, (2) presumed positive, (3) presumed negative and (4) confirmed negative. Premises that were classified as PED confirmed positive were premises that had confirmed positive real-time reverse, transcriptase polymerase chain reaction (RT-PCR) test for PEDV at the Animal Health Laboratory (AHL) at the University of Guelph. A presumed positive status was declared based on pig flow and movement as identified by the premises' veterinarian and did not require any diagnostic testing. Thus, premises that housed animals that were sourced from a PED-positive premises were classified as presumed positive due to movement of presumed infected pigs. Presumed negative premises were previously positive premises (i.e., either previously confirmed or presumed positive), where the producer implemented measures to eliminate PEDV from the herd and confirmed the virus to be eliminated through animal or environmental testing. Sampling methods for classifying premises as presumed negative were based on herd type and pig flow, and considered different types of samples (i.e., individual swabs, Swiffer samples, oral fluids, etc). The basic considerations for all sampling types were: 98% individual test sensitivity, 100% individual test specificity, maximum design prevalence of 10%, and 95% confidence in detection of disease at the design prevalence level (9). Lastly, premises that were classified as confirmed PED negative were premises in which there were no clinical or diagnostic evidence of PED for at least 6 months after the presumed negative status update.

### Descriptive Analysis

Data was entered into Microsoft Excel Version 16.14.1 (Microsoft, Redmond, Washington, USA) and then imported into Stata Version 13.1 (StataCorp, College Station, Texas, USA). The proportion of premises that were confirmed PED-positive, presumed PED-positive and presumed PED-negative by herd type were documented. Also, the proportion of herds to eliminate PEDV by herd type, season and the year of PEDV diagnosis were recorded. The median time to elimination and the 25th percentile, along with 95% confidence intervals (CI) were estimated by herd type, season and the year of PEDV diagnosis.

### Statistical Analysis

The DCP database consisted of 144 confirmed or presumed PED-positive case herds. Four herds reported subsequent infections, which were excluded from further analysis. In addition, one herd was excluded because the herd type was unknown and another herd was excluded because it was categorized as an isolation/acclimatization unit. Therefore, 138 confirmed or presumed PED-positive case herds were included in the study. A binary variable was created to indicate whether the case herds eliminated PEDV (censored = 1) off-site during the study duration and if the herds did not eliminate PEDV (censored = 0) during the study duration or due to loss-to-follow-up (censored = 0). For the case herds that did not report a change in the virus status over the study period of interest (*n* = 8), the herds were considered to be censored at times when their observation period ended. Similarly, there were cases (*n* = 14) that reported a change in infection status change that was >100 weeks (~2 years) after the initial date of infection. These herds were censored at 100 weeks. Consequently, a total of 22 herds had their time censored and 116 herds had the event of interest (i.e., reported to have eliminated the virus at least 10% level with 95% confidence).

The time taken to eliminate PEDV from participating premises were estimated using Kaplan-Meier survival curves by herd type, season of diagnosis and year of diagnosis. The variable season was computed and based on northern meteorological seasons. Winter was defined as any confirmed or presumed PEDV diagnosis between December 1st and February 28th, as well as February 29th for the year of 2016 to account for the leap year (10). Any confirmed or presumed PEDV diagnosis between March 1st and May 31st, June 1st and August 31st and, September 1st and November 30th were classified into the variable season as Spring, Summer, and Fall, respectively (10). Log-ranked tests were computed for the 3 categories of Kaplan-Meier survival curves (herd type, year of diagnosis and season of diagnosis).

A Cox's proportional hazard model was constructed to investigate the effect of explanatory variables including herd type, season of diagnosis and year of diagnosis on the time to eliminate PEDV from the premises. The time to event (i.e., elimination) was identified as the time in weeks for a premises to change from confirmed or presumed PED-positive to presumed PED-negative. A failure occurred if the premises eliminated PEDV. Univariable analysis was done using the 3 predictor variables mentioned above, separately. The multivariable model was built using a manual forward selection procedure, with a *p* < 0.10, based on a partial likelihood ratio test as an inclusion criterion. The assumption of the Cox's proportional hazard model was evaluated graphically showing the logarithm of the estimated cumulative hazard function. Goodness-of-fit was evaluated using a Hosmer-Lemeshow test and a Harrell's C concordance statistic. Deviance and score residuals were evaluated.

## Results

### Descriptive Analysis

From January 2014 to October 2017, a total of 138 PED cases were reported in the DCP database. From the participating premises in the DCP database, 60.1% were finisher sites (*n* = 83), 11.6% were nursery sites (*n* = 16), 10.2% were farrow-to-finish (*n* = 14), 10.2% were farrow-to-wean (*n* = 14), 4.3% were wean-to-finish (*n* = 6) and 3.6% were farrow-to-feeder (*n* = 5), respectively.

Ninety-four cases (65.2%, 90/138) reported that they were confirmed PED-positive. Of these 90 cases, 92.2% (*n* = 83) reported that they eliminated PEDV and therefore gained a presumed-negative status. Forty-eight cases (34.8%, 48/138) reported that they were initially presumed PED-positive, at their initial date of infection. Of these 48 cases, 97.8% (*n* = 47) reported that they eliminated PEDV during the study period and achieved a presumed-negative status.

Kaplan-Meir estimates of the median and 25th percentile time in weeks to eliminate PEDV are displayed in Table 1. Nursery herds had the shortest median (23 weeks, 95% CI: 14, 31) and 25th percentile (16 weeks, 95% CI: 1, 23) for the duration of time it took in weeks to eliminate PEDV. Farrow-to-feeder herds had the longest median time (43 weeks 95% CI: 18, NA) and second longest 25th percentile (27 weeks, 95% CI: 18, 79) for the amount of time it took in weeks to eliminate PEDV. Cases that were diagnosed in the spring and winter seasons had higher medians and 25th percentiles for the amount of time it took in weeks to eliminate PEDV compared to cases that were diagnosed in fall and summer seasons (Table 1). The median time to PEDV elimination in swine herds infected in 2014, 2015, 2016, and 2017 were 30, 34, 32, and 18 weeks, respectively (Table 1).

**Table 1:** Descriptive statistics^1^ using a disease control program database^*^ to determine the median and 25th percentile amounts of time it took, in weeks, to eliminate porcine epidemic diarrhea virus.

**Variable**	**Number of premises**	**Median**	**95% CI**	**25th percentile**	**95% CI**
**Herd type:**					
Farrow-to-wean	13	25	(16, 31)	18	(2, 25)
Wean-to-finish	4	42	(32, NA)	32	(32, 55)
Farrow-to-finish	15	32	(16, 47)	24	(10, 31)
Finisher only	84	33	(30, 38)	25	(21, 28)
Nursery only	17	23	(14, 31)	16	(1, 23)
Farrow-to-feeder	5	43	(18, NA)	27	(18, 79)
**Season:**					
Winter	46	34	(31, 38)	29	(25, 32)
Spring	58	37	(28, 42)	24	(20, 28)
Summer	26	24	(17, 28)	16	(3, 22)
Fall	8	11	(1, 36)	1	(1, 16)
**Year:**					
2014	92	30	(26, 35)	23	(20, 25)
2015	27	34	(30, 64)	24	(12, 33)
2016	16	32	(21, 44)	21	(3, 32)
2017	3	18	(13, NA)	13	(13, NA)

* *The Area Regional Control and Elimination program (ARC&E) database was used to collect diagnostic data on porcine epidemic diarrhea virus herd status of Ontario swine herds on a weekly basis using the Animal Health Laboratory (AHL) at the University of Guelph. Premises that volunteered to participate in the program from January 2014 to October 2017 were included in the study. Descriptive survival analysis statistics are described above*.

*CI, confidence interval; SE, standard error*.

1 *Kaplan-Meier survival curves by herd type, season of diagnosis and year of diagnosis were created. Kaplan-Meir estimates of the median and 25th percentile time in weeks to eliminate porcine epidemic diarrhea virus was calculated*.

### Statistical Analysis

**Figure 1:**
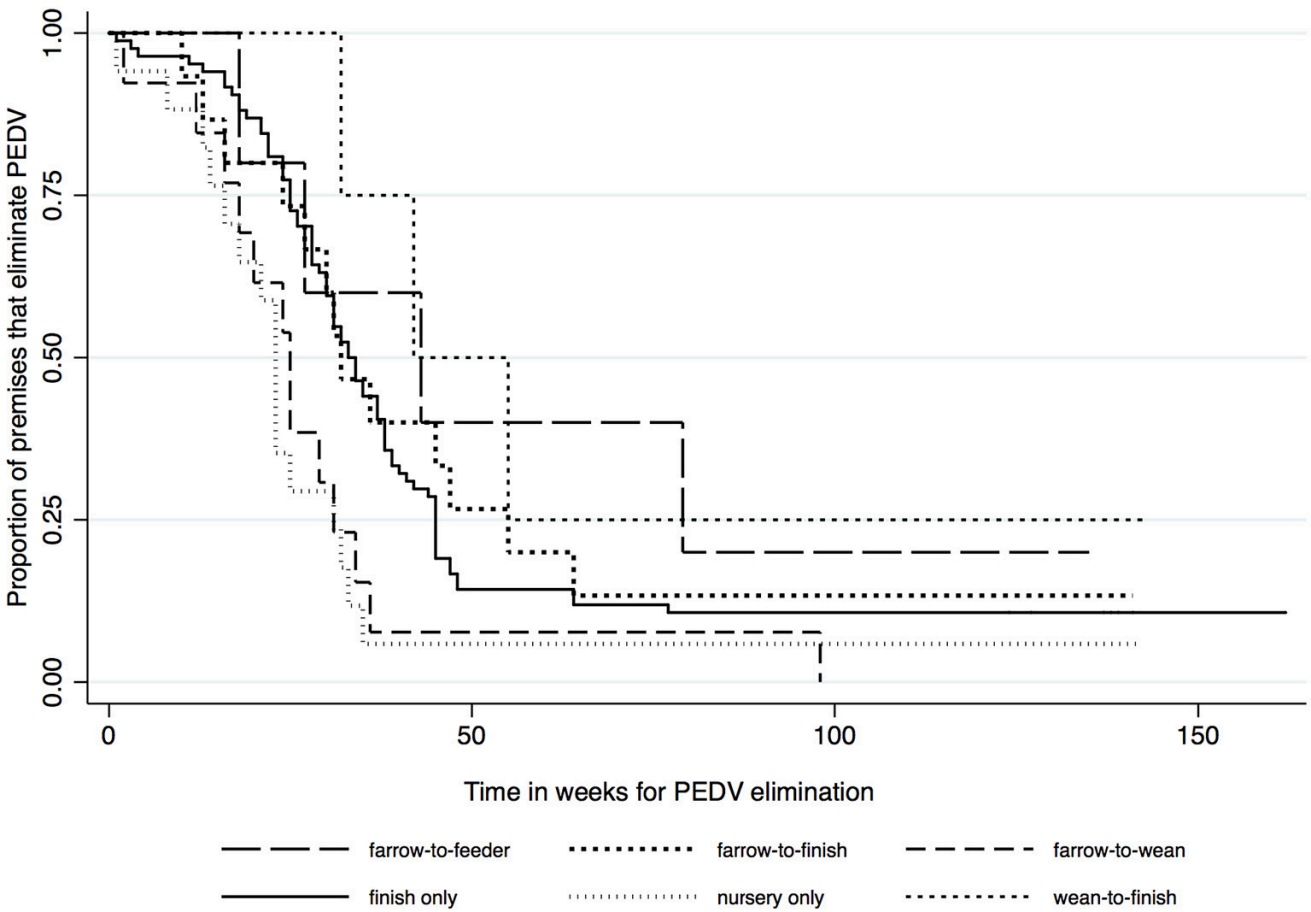
Kaplan-Meier survival functions based on herd type using the porcine epidemic diarrhea virus disease control program database^*^. ^*^The Area Regional Control and Elimination program (ARC&E) database was used to collect diagnostic data on porcine epidemic diarrhea virus herd status of Ontario swine herds on a weekly basis using the Animal Health Laboratory (AHL) at the University of Guelph. Premises that volunteered to participate in the program from January 2014 to October 2017 were included in the study.

**Figure 2:**
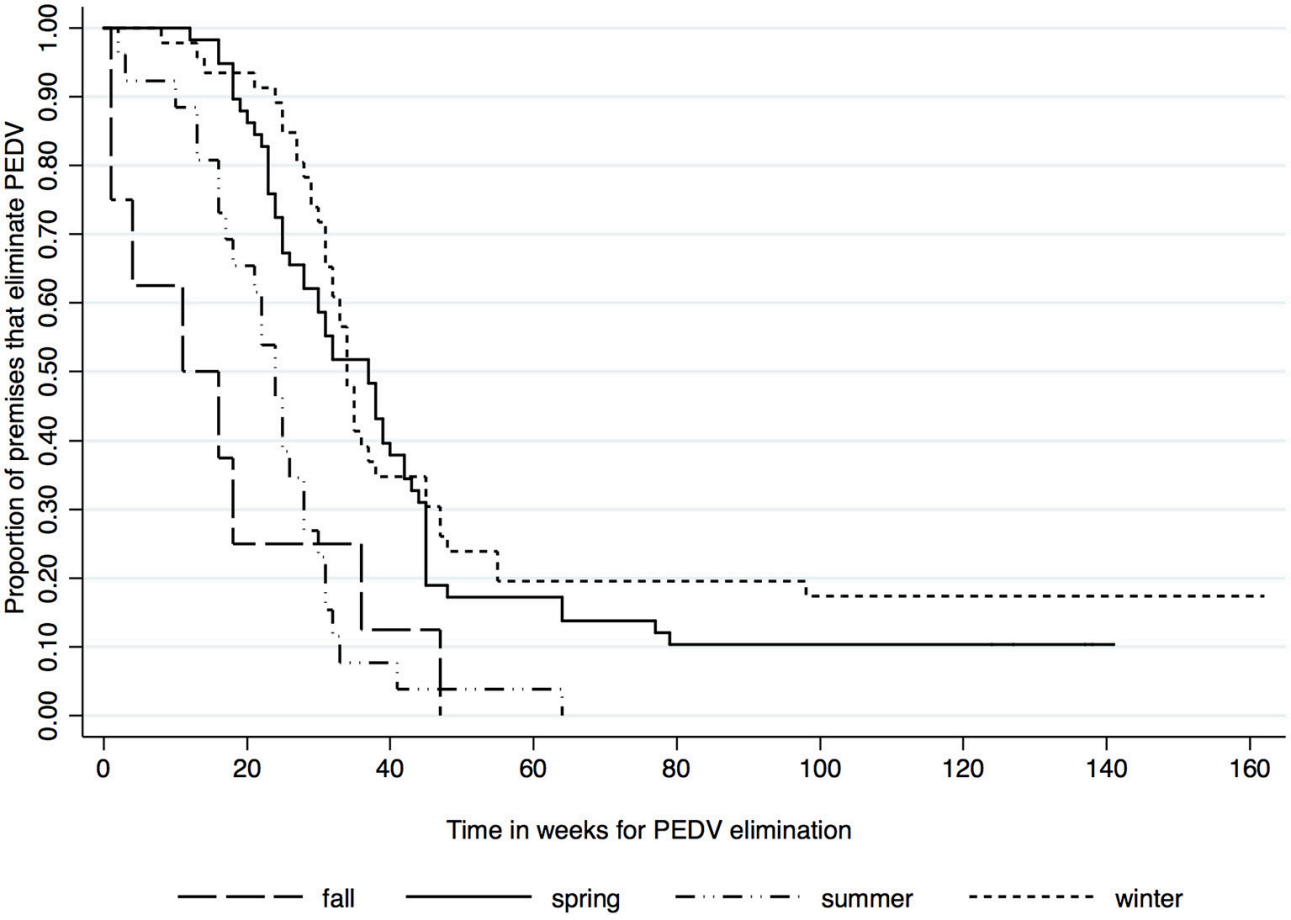
Kaplan-Meier survival functions based on season of diagnosis using the porcine epidemic diarrhea virus disease control program database^*^. ^*^The Area Regional Control and Elimination program (ARC&E) database was used to collect diagnostic data on porcine epidemic diarrhea virus herd status of Ontario swine herds on a weekly basis using the Animal Health Laboratory (AHL) at the University of Guelph. Premises that volunteered to participate in the program from January 2014 to October 2017 were included in the study.

**Figure 3:**
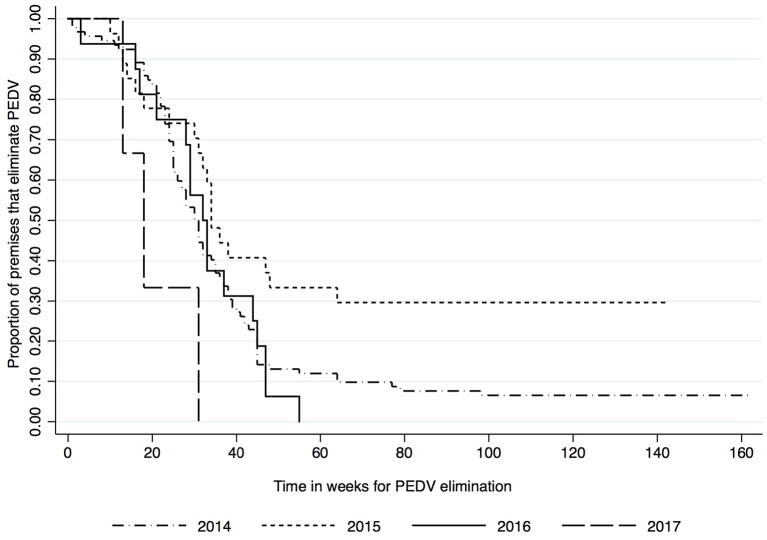
Kaplan-Meier survival functions based on year of diagnosis using the porcine epidemic diarrhea virus disease control program database^*^. ^*^The Area Regional Control and Elimination program (ARC&E) database was used to collect diagnostic data on porcine epidemic diarrhea virus herd status of Ontario swine herds on a weekly basis using the Animal Health Laboratory (AHL) at the University of Guelph. Premises that volunteered to participate in the program from January 2014 to October 2017 were included in the study.

Kaplan-Meier survival functions based on herd type, season and year of diagnosis are presented in Figures 1–3. The log-rank test statistic evaluating the equality of survival functions between herd types was statistically significant (*p* = 0.0029). Similarly, the season a premises was declared as PEDV-positive (*p* < 0.001) and the year of initial PEDV confirmation (*p* = 0.0105) were both statistically significant.

The results of the univariable analyses conducted through Cox's proportional hazard model are reported in Table 2. Briefly, herd type (*p* = 0.011), season (*p* < 0.001), and year of initial diagnosis (*p* = 0.019) were all associated with the likelihood of elimination in univariable analyses. The final multivariable model also included herd type, season of diagnosis and year of diagnosis and is presented in Table 3.

**Table 2:** Results of univariable analyses^1^ using a disease control program database^*^ to determine the hazard ratios associated with herd type, season of diagnosis, and year of diagnosis with the amount of time it took, in weeks, to eliminate porcine epidemic diarrhea virus.

**Variable**	**Hazard ratio**	***p*-value**	**Overall *p*-value**	**95% CI**
**Herd type:**				
Farrow-to-feeder^†^				
Farrow-to-wean	2.98	0.058	0.011	0.97, 9.20
Wean-to-finish	0.77	0.73		0.17, 3.45
Farrow-to-finish	1.31	0.64		0.43, 4.03
Finisher only	1.46	0.46		0.53, 3.99
Nursery only	3.35	0.032		1.11, 10.10
**Season:**				
Winter^†^				
Spring	1.20	0.40	<0.001	0.79, 1.82
Summer	3.26	0.001		1.95, 5.45
Fall	3.57	<0.001		1.65, 7.69
**Year:**				
2014^†^				
2015	0.54	0.015	0.019	0.33, 0.89
2016	1.07	0.80		0.63, 1.83
2017	2.91	0.072		0.91, 9.32

* *The Area Regional Control and Elimination program (ARC&E) database was used to collect diagnostic data on porcine epidemic diarrhea virus herd status of Ontario swine herds on a weekly basis using the Animal Health Laboratory (AHL) at the University of Guelph. Premises that volunteered to participate in the program from January 2014 to October 2017 were included in the study. Descriptive survival analysis statistics are described above*.

† *Referent categories*.

*SE, standard error; CI, confidence interval*.

1 *A Cox's proportional hazard model was constructed to investigate the effect of several predictor variables including herd type, season of diagnosis and year of diagnosis upon the time to eliminate PEDV from the premises in 3 univariable models. The time to event (i.e., elimination) was identified as the time in weeks for a premises to change from confirmed or presumed PED-positive to confirmed or presumed PED-negative. A failure occurred if the premises eliminated PEDV*.

**Table 3:** Results of multivariable analyses^1^ using a disease control program database^*^ to determine the hazard ratios associated with herd type, season of diagnosis, and year of diagnosis with the amount of time it took, in weeks, to eliminate porcine epidemic diarrhea virus.

**Variable**	**Hazard ratio**	***p*-value**	**95% CI**	**Partial likelihood ratio (*p*-value)**
**Herd type:**				
Farrow-to-feeder^†^				
Farrow-to-wean	3.36	0.044	1.03, 10.93	<0.001
Wean-to-finish	0.61	0.53	0.13, 2.83	
Farrow-to-finish	0.80	0.70	0.25, 2.54	
Finisher only	1.07	0.89	0.39, 2.98	
Nursery only	2.33	0.15	0.74, 7.36	
**Season:**				
Winter^†^				
Spring	1.40	0.20	0.84, 2.31	<0.001
Summer	5.04	<0.001	2.74, 9.27	
Fall	7.32	<0.001	3.12, 17.18	
**Year:**				
2014^†^				
2015	0.42	0.002	0.25, 0.72	<0.001
2016	1.62	0.10	0.91, 2.89	
2017	2.15	0.21	0.64, 7.15	

* *The Area Regional Control and Elimination program (ARC&E) database was used to collect diagnostic data on porcine epidemic diarrhea virus herd status of Ontario swine herds on a weekly basis using the Animal Health Laboratory (AHL) at the University of Guelph. Premises that volunteered to participate in the program from January 2014 to October 2017 were included in the study. Descriptive survival analysis statistics are described above*.

† *Referent categories*.

*SE, standard error; CI, confidence interval*.

1 *A Cox's proportional hazard model was constructed to investigate the effect of several predictor variables including herd type, season of diagnosis and year of diagnosis upon the time to eliminate PEDV from the premises in a multivariable model. The time to event (i.e., elimination) was identified as the time in weeks for a premises to change from confirmed or presumed PED-positive to confirmed or presumed PED-negative. A failure occurred if the premises eliminated PEDV. In the current study, the Hosmer-Lemeshow test indicated that the model fits the data (p = 0.46). Also, Harrell's C concordance statistic computed (0.72) found that the model had good overall predictive ability*.

Farrow-to-wean premises were 3.36 times more likely than farrow-to-feeder herds (referent category) to eliminate the virus throughout the study period (Table 3). The hazard ratio for premises diagnosed in the summer and fall months was 1.40 (*p* < 0.001, 95% CI: 2.74, 9.27) and 7.32 (*p* < 0.001, 95% CI: 3.12, 17.18), respectively. Thus, premises that were diagnosed in the summer and fall months were more likely than herds diagnosed in winter months (referent category) to eliminate PEDV. Premises that were diagnosed with PEDV in 2015, had a hazard of eliminating PEDV that was 0.54 times the hazard of eliminating PEDV in herds diagnosed with PEDV in 2014 (*p* = 0.015, 95% CI: 0.33, 0.89). This suggests that herds that were diagnosed with PEDV in 2015 were less likely to eliminate the virus compared to premises that were diagnosed in 2014 (referent category). In contrast, premises that were diagnosed in 2016 were 1.62 times more likely to eliminate the virus compared to herds diagnosed in 2014 (*p* = 0.10, 95% CI: 0.91, 2.89).

The assumption of the Cox's proportional hazard model was examined graphically showing the logarithm of the estimated cumulative hazard function. There was no indication that the season of diagnosis and herd type variables had a time varying effect and therefore, the assumption of proportional hazards was met. The Hosmer-Lemeshow test indicated that the model fits the data (*p* = 0.46). Also, Harrell's C concordance statistic computed (0.72) found that the model had good overall predictive ability. There were no outliers or influential observations found.

## Discussion

Following the emergence of PEDV into the United States in 2013, many actions were taken in Ontario in anticipation of the emergence of the virus into Ontario. Newsletters, producer meetings and advertisements were communication tools that were used to inform producers of the risk of PED entry and to elaborate on prevention strategies (11). Following the initial emergence, the outbreak in the province of Ontario was well controlled, which was achieved through quick identification of the suspected source of outbreak and implementation of biosecurity practices aimed to prevent further spread of infection. This resulted in a relatively low prevalence of infected herds (7), which could have contributed to willingness to eliminate PEDV infection. Veterinarians have implemented site-specific elimination strategies in Ontario, however the duration of time for a premises to eliminate the virus is variable based on a multitude of factors (i.e., the initial start time for the elimination process may depend on the PEDV status of the sow herd, or the season). The starting time for the time to elimination in this study was not the start date of control measures aimed at elimination, but the date of original infection. In part, due to this reason, the median time to elimination was relatively long. However, we believe that this time to elimination gives veterinary authorities reasonable overview of time to elimination for a newly emerging disease in the area, for which previous experience in elimination did not exist.

An important finding in this study is that with the exception of 2015, the estimated hazard of eliminating PEDV increased over the years examined. Although exact reasons are difficult to determine, it is possible that a combination of factors played a role. Veterinary practitioners were initially dealing with a new emerging disease into Canada, and it is possible that they developed more expertise in procedures to eliminate PED from herds as time went on. Additionally, most cases occurred during the first 2 years (*n* = 92 in 2014 and *n* = 27 in 2015) of the outbreak and it is possible that resources needed to be prioritized between actions needed to prevent further spread and actions to eliminate infection from already infected sites, particularly if such sites required substantial planning. In contrast, the number of new cases in 2016 (*n* = 16) and 2017 (*n* = 3) was substantially lower.

Another important finding in this study was that herds diagnosed in winter and spring months required more time to eliminate the virus. This was likely due to PEDV's survivability and ability to remain infectious. Typically, coronaviruses can survive temperatures from 56°C for 10–15 mins, 37°C for several days, 4°C for several months, and while frozen at −60°C many years without losing infectivity (12). Thus, it was likely difficult to eliminate the virus due to its survivability in Ontario's temperatures in the spring and winter months. It is also possible that due to the lack of external pressures, producers who had positive herds waited until warmer months to start with the PEDV elimination protocol.

Farrow-to-wean herds were found to eliminate the virus in a shorter amount of time compared to farrow-to-feeder herds. This was an expected finding, since in a farrow-to-wean operation; the system is generally less complex than a farrow-to-feeder or farrow-to-finish operation. For instance, farrow-to-wean herds have fewer types of production classes than farrow-to-finish herds. The presence of nursery pigs on the same site as suckling pigs complicates infection control practices since a separate set of control measures and operating procedures need to be designed and implemented for the nursery stage of production. This requires resources, strict adherence to internal biosecurity protocols and often demographic measures, such as creation of an interruption, or gap, in pig flow. Pig flow through a production system, and more specifically, the creation of a gap in pig flow, is now recognized as an essential aspect of achieving earlier farrowing site elimination by allowing more effective cleaning and disinfection protocols required for successful elimination (13). Pig flow through a production system is the frequency of introducing new pigs into a population and the amount of opportunity these pigs have to come in contact with other pigs. A gap in pig flow however is often a one-time event to prevent the entrance of new animals to control the spread of the virus. A partial depopulation could present a gap in pig flow, where infected animals are removed from the herd, followed by cleaning and decontaminating the site. The database did not include details about specific infection control practices, such as the details of pig flow or attempts to generate a gap in pig flow. Nonetheless, it is also worth pointing out that the variability in the time to elimination was markedly higher in farrow-to-feeder than in other herd types. It is possible that this time to elimination is not only driven by herd demographics and pig flow, but also with other factors such as willingness to eliminate, which was not directly measured in this study.

An important concept for this study is that the data collected was from a large-scale industry-based surveillance program. This study does present limitations. Firstly, the DCP is based on voluntary participation. The Animal Health Act in Ontario required that all PED-positive herds report to the Ontario Ministry of Agriculture, Food and Rural Affairs (OMAFRA), by law, when the hazard was deemed emerging. The OMAFRA surveillance program only accounts for primary case herds, which are case herds with a positive diagnostic test (RT-PCR) for PEDV (14). Thus, secondary cases due to animal movement were not included in the OMAFRA surveillance program. Unlike the surveillance program managed by OMAFRA, where 100% coverage of primary PEDV-infected cases were included, the DCP used in the study only includes primary case herds that volunteered to participate in the program, and secondary cases resulting from animal movement from such cases (7).

It is also possible that some producers or veterinarians did not follow up to report that the case indeed eliminated PEDV from the premises, in which case the estimated time to elimination would be longer than in reality. There were 14 premises for which the records indicated that the time between initial infection and a change in status to presumed negative was longer than 100 weeks. The survival time of these premises was censored at 100 weeks. Since participation in this large-scale disease monitoring program is not mandatory, it is possible that some of these premises were not working toward eliminating the virus, since there was no external pressure to do so. Alternatively, it is likely that owners that had a low prevalence of PEDV on-site, may have not tested pigs to confirm PEDV status (i.e., the absence from infection). However, both of these scenarios could occur with a production-limited disease. If large-scale disease control programs are initiated at the level that is different than a premises, or production-system level; veterinary authorities should be aware of the situations where time to negativity could take a long time. In addition, populations with high replacement and/or birth rates such as swine herds could have considerable number of susceptible animals introduced into a population that is partially immune due to recent exposure. This situation could provide opportunity for infectious agents to continue circulating at low levels. Consequently, declaring freedom from infection at 10% may not be sufficient. However, making a decision about the design prevalence should be weighed against the disease epidemiology and cost to producers. Another limitation is the database was missing variables for herd size. Due to this, the authors decided not to consider this variable in the analysis. However, despite these limitations, this study provided novel insight in regards to PEDV elimination times in Ontario.

In conclusion, this study allowed estimation of time to PEDV elimination based on a large-scale disease control program database, which considered time between initial infection and confirmation of PEDV freedom at a minimum level of 10%. Under such assumptions, the median time to elimination of PEDV from Ontario swine herds varied between 23 weeks in nursery herds (standard error = 1 week), and 43 weeks (standard error = 17.5 weeks) in farrow-to-feeder herds. Herd type, season, and year of original diagnosis were all associated with the time to negativity (*p* < 0.05) in the multivariable model. Among the sow herds, farrow-to-wean herds had the highest hazard of PEDV elimination. These results are reflective of the complexity of the infection control practices applied in herds with different demographics and population structures. The hazard of elimination was also higher in herds that had the initial infection during summer and fall than in herds that had the initial infection during winter. This could be a reflection of seasonal variability in the pathogen transmissibility or decisions made at the level of individual herds to proceed with infection control measures when the likelihood of success is the highest. With the exception of the second year after initial emergence, the hazard of elimination increased over years, which could reflect the availability of resources to manage an emerging production-limiting disease. The median time to elimination was relatively long in all herd types. However, this could be a consequence of the way it was measured, the decisions about implementation of infection control measures which could be made at the level of individual herds, multi-site production systems or possibly delays related to reporting PEDV elimination. Nonetheless, the design of control measures for production-limiting diseases at the regional level should consider these factors.

## Ethics Statement

Research Ethics Board of the University of Guelph approved the study. Data for this study were obtained from Swine Health Ontario under an appropriate data sharing agreement based on secondary data usage.

## Author Contributions

AP conducted data management, analysis, and interpretation of results under guidance of ZP and TO. Manuscript was written by AP, with input from ZP, TO, CD, and JH.

### Conflict of Interest Statement

The authors declare that the research was conducted in the absence of any commercial or financial relationships that could be construed as a potential conflict of interest.
